# Endocytoscopic observation with methylene blue staining for duodenal neoplasms associated with familial adenomatous polyposis

**DOI:** 10.1038/s41598-020-76309-6

**Published:** 2020-11-05

**Authors:** Youichi Kumagai, Eisuke Yamamoto, Morihiro Higashi, Toru Ishiguro, Satoshi Hatano, Yoshitaka Toyomasu, Kunihiko Amano, Okihide Suzuki, Kei-ichiro Ishibashi, Erito Mochiki, Jun-ichi Tamaru, Hideyuki Ishida

**Affiliations:** 1 Department of Digestive Tract and General Surgery, Saitama Medical Center, Saitama Medical University, 1981 Kamoda, Kawagoe, Saitama 350-8550 Japan; 2 Department of Pathology, Saitama Medical Center, Saitama Medical University, Saitama, Japan

**Keywords:** Gastrointestinal cancer, Cancer, Gastroenterology, Oesophagogastroscopy

## Abstract

Duodenal cancer is a leading cause of death after colectomy in patients with familial adenomatous polyposis (FAP). Detailed endoscopic evaluation of duodenal lesions with potential for carcinoma development is therefore mandatory. Here we investigated the features of duodenal lesions in FAP patients using an endocytoscopy system (ECS). We retrospectively reviewed duodenal lesions in 15 cases of FAP using an ECS (GIF-H290EC) with methylene blue (MB) as the vital dye. With reference to the Spigelman classification, we investigated the number of lesions using white light (WL), narrow-band imaging (NBI), and MB staining. Using the maximum magnification power of the ECS we investigated the histology (duct openings or finger-like projections) and grade of dysplasia (presence or absence of enlarged oval-shaped nuclei) of the lesions. The number of duodenal lesions increased in ascending order of WL, NBI, and MB (P < 0.05). Among 51 MB-unstained lesions, 46 (90.2%) were proven to be duodenal neoplasms histologically. Duct openings were seen in 90.2% of tubular adenomas and tubulovillous adenomas. Finger-like projections were seen in 33.3% of tubular adenomas and in 88.2% of tubulovillous adenomas. Enlarged oval-shaped nuclei were observed in 100% of duodenal cancers, 33.3% of high-grade adenomas, and 9.4% of low-grade adenomas. MB staining allows more accurate detection of duodenal neoplasms in comparison to conventional WL and NBI observation. In cases of FAP, use of the maximum magnification power of the ECS may allow selection of lesions with high malignant potential.

## Introduction

Familial adenomatous polyposis (FAP) is an autosomal dominant cancer predisposition syndrome caused by germline mutation in the adenomatous polyposis coli (APC) gene^1^. It is a rare disease, with an incidence of roughly 1 in 10,000–20,000 live births^2^. Patients with FAP develop numerous adenomas of the colon and invariably develop carcinoma. In addition, the duodenum is the second most common site for polyps in FAP. The lifetime risk of duodenal adenoma for FAP patients is estimated to be more than 90%^3^, and 3–12%^4^ of such patients develop duodenal cancer, which is one of the main causes of death^5^. Therefore, detailed endoscopic surveillance of duodenal polyposis with the potential to develop into carcinoma is mandatory.

Spigelman et al.^6^ published a landmark study of the risk factors for development of duodenal carcinoma in FAP patients. Spigelman’s original classification was partially modified with reference to the Vienna classification^7^, and categorized patients with duodenal polyps into Stages 0-V based on the number of polyps, polyp size, pathology (tubular, tubulo-villous, or villous), and the degree of dysplasia (low grade or high grade). Narrow-band imaging (NBI), a form of image-enhanced endoscopy, allows detection of more adenomatous lesions in comparison with conventional white light (WL) endoscopy^8^. However, it remains unclear whether NBI or NBI magnifying endoscopy can allow predictive histological diagnosis of duodenal lesions^9^, and for this purpose histopathological examination of biopsy samples remains the mainstay.

The endocytoscopy system (ECS) is a novel ultra-high-magnification endoscope that can visualize surface epithelial cells^10^. We have previously reported the features of normal duodenal mucosa, duodenal adenoma and non-ampullary mucosal duodenal cancer revealed by ECS^11,12^. Nuclei in normal duodenal mucosa were found to show a regular arrangement in each villus and goblet cells were sparse, whereas adenomatous lesions showed loss of goblet cells and spindle-shaped nuclei with loss of polarity. Furthermore, round duct openings were observed in tubular adenoma, and finger-like projections were evident in villous adenoma. A characteristic feature of duodenal cancer when observed by ECS is the presence of enlarged oval-shaped nuclei.

If it were possible to detect duodenal cancers or high-grade adenomas at an early stage, endoscopic resection of these lesions and preservation of the duodenum might be feasible. In the present study, we employed the ECS for investigating the microscopic appearance of duodenal lesions in FAP patients using methylene blue (MB) staining in vivo.

## Materials and methods

### Equipment

We employed the GIF-H290EC instrument for this study. This ECS has one lens whose magnification can be increased consecutively from the conventional endoscopy level through to the NBI-magnification endoscopy level (approximately 100×), and to a maximum of 500× (tissue field of view, 570 × 500 μm) using a hand lever.

### In vivo observation

Between June 2018 and April 2020, we used ECS to examine the upper gastrointestinal tract of 15 FAP patients who were scheduled for periodic surveillance (11 patients) or elective duodenectomy (4 patients). The patients comprised 5 females and 10 males with a median age of 43 (range, 26–83) years.

The preparations for ECS observation, including pharyngeal anesthesia, were similar to those used for conventional endoscopy. Conscious sedation was performed using intravenous midazolam. We also tried to exclude the influence of peristalsis using intravenous scopolamine butylbromide. Using the GIF-H290EC, we first carried out non-magnified conventional WL observation followed by NBI observation with or without slight magnification (approximately 100×), and screened the entire duodenal mucosa. We then sprayed 2–4 ml of 1% or 2% MB as a vital dye onto the surface of the entire duodenal mucosa. Approximately 1 min after spraying the dye, we washed the duodenal mucosa with water and observed the duodenum without magnification. We then brought the ECS lens into contact with the lesion and finally zoomed up to the maximum magnification. After ECS observation we took biopsy samples from the lesion. We observed the duodenal lesions using the maximum magnifying power as far as possible. In cases of insufficient vital staining, re-staining was performed. After the biopsy specimens had been obtained, all of the lesions were examined histologically. The median duration of the endoscopic procedure including stomach observation was 24 (18–32) minutes.

### Data evaluation

**Figure 1:**
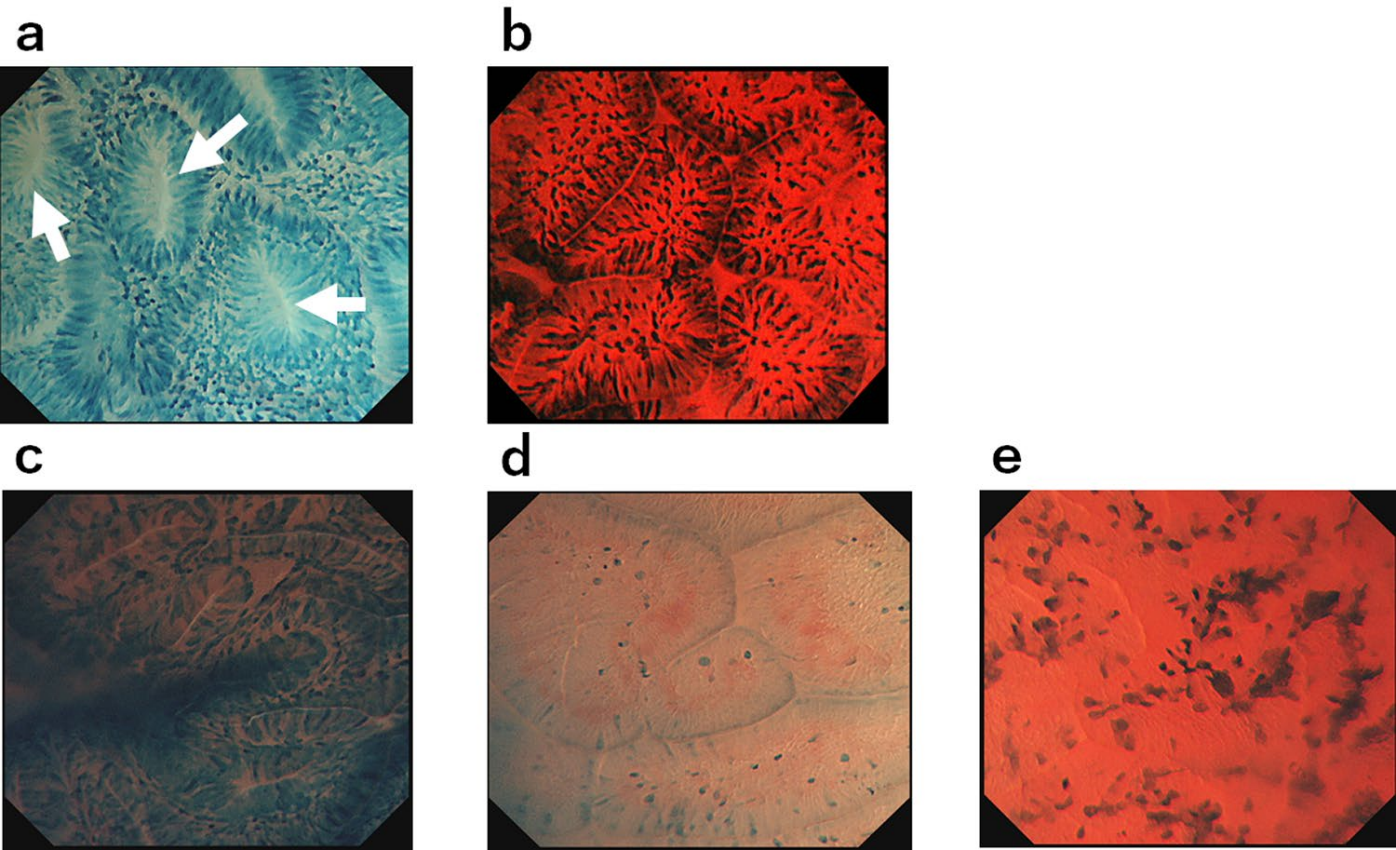
Microstructure of the duodenal lesions observed using the endocytoscopy system. All pictures were obtained using the maximum magnification power of the ECS (×500). (**a**) Slit-like duct opening (arrow) with circularly arranged epithelial cells, which is a feature of tubular adenoma. (**b**) Triangular or rectangular shaped finger-like projection, i.e. a pointed villous structure, which is a feature of villous adenoma. (**c**) Spindle-shaped nuclei without nuclear enlargement. (**d**) Oval-shaped nuclei without nuclear enlargement. (**e**) Prominent enlarged oval-shaped nuclei with spindle-shaped nuclei.

From the recorded video, two independent endoscopists counted the number of duodenal lesions and classed them into 4 categories (0: 0, 1: 1–4, 2: 5–20, 3: > 20) under WL observation, NBI observation, and after MB staining, respectively. Two endoscopists who were not aware of the final histological diagnosis reviewed the ECS pictures of each lesion and determined the microstructure [presence or absence of duct openings or finger-like projections (Fig. 1a,b)] and nuclear shape [presence or absence of nuclear enlargement, and spindle-shaped nuclei only or intermixed with oval-shaped nuclei (Fig. 1c–e)] by mutual consensus.

### Statistical analysis

Variables were expressed as the median (range). Kappa values were calculated to assess the degree of inter-observer agreement. Differences between related groups were analyzed by Tukey’s test at a significance level of *P* < 0.05.

All procedures performed in studies involving human participants were in accordance with the ethical standards of the institutional and/or national research committee and with the 1964 Helsinki Declaration and its later amendments or comparable ethical standards.

Written informed consent was obtained from all patients, and this study was approved by the hospital ethics committee at Saitama Medical Center, Saitama Medical University (reference number: 1336-II).

## Results

### Non-magnified endoscopic view and number of lesions

**Figure 2:**
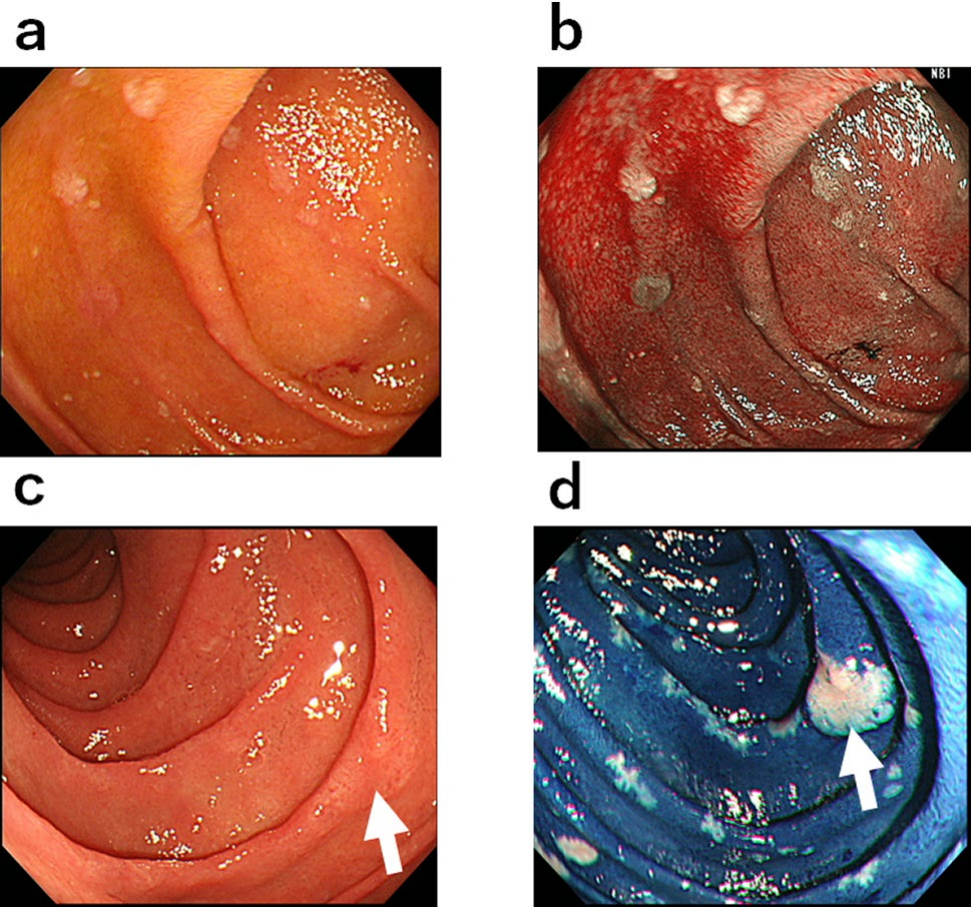
Non-magnified endoscopic appearance under white light, narrow-band imaging, and methylene blue staining. (**a**) White light endoscopic observation of a FAP patient. Whitish or normal-colored tiny protruding lesions are evident. (**b**) Same lesion as that in (**a**) observed by narrow-band imaging. Neoplastic lesions are enhanced as whitish or brownish protruding lesions more clearly in comparison to white light observation. (**c**) White light endoscopic observation. One slightly whitish and elevated lesion is evident (arrow). (**d**) Same lesion as that in (**c**) after methylene blue staining. In addition to the elevated lesion in (**c**) (arrow), multiple unstained areas show a “cloud in blue sky” appearance.

Under WL observation, the non-magnified view depicted duodenal neoplasms as whitish and slightly elevated lesions, even those less than 1 mm in diameter (Fig. 2a). NBI demonstrated these lesions as whitish and clearly enhanced from the background brownish normal duodenal mucosa (Fig. 2b). Some depressed lesions were recognized as more brownish relative to the normal duodenal mucosa. After MB staining, the normal duodenal mucosa was stained dark blue, and this enhanced the whiteness of the neoplastic lesions, giving a “cloud in blue sky” appearance (Fig. 2c,d).

The scores for the number of duodenal lesions counted by endoscopist 1 are shown in Table 1. The use of NBI increased the score (1 rank) for 8 of the 15 cases in comparison to WL observation, and after MB staining the score was increased for 7 of the 15 cases (6 cases: 1 rank, 1 case: 2 ranks) relative to NBI observation. The differences between the groups (WL vs. NBI and NBI vs. MB) were significant (P < 0.05). Kappa values for comparison between endoscopists 1 and 2 were 0.71, 0.81, and 0.89, (P = 0.00) for WL, NBI, and MB observation, respectively.

**Table 1:**
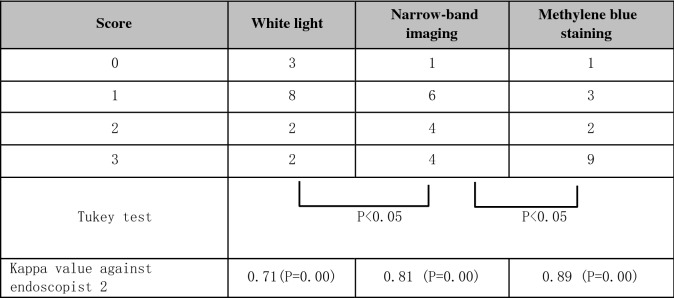
Scores for the numbers of duodenal lesions made by endoscopist 1.

Score 0: 0 lesion, Score 1: 1–4 lesions, Score 2: 5–20 lesions, Score 3: > 20 lesions.

### Histological evaluation using ultra-high magnification with the ECS

We carried out random observations using cellular level magnification with the ECS and confirmed the biopsy histology in a total of 51 duodenal lesions (44 lesions examined by biopsy, and 7 after surgical resection) from the 15 patients (median 3 lesions (0–7) per patient). Of these lesions, 46 (90.2%) were diagnosed as duodenal neoplasms histologically on the basis of biopsy or surgical resection. Five of the lesions were duodenal cancers (one Vater papilla cancer and four non-ampullary mucosal cancers) and 41 were duodenal adenomas (9 high-grade adenomas, 32 low-grade adenomas, 17 tubulovillous adenomas and 24 tubular adenomas). The median maximum diameters of the low-grade adenomas, high-grade adenomas and duodenal cancers were 5 mm (2–12 mm), 7 mm (3–40 mm), and 10 mm (7–50 mm), respectively. With regard to macroscopic appearance, the flat type was the dominant type of adenomatous lesion (23/32 (71.9%) of low-grade adenomas and 5/9 (55.6%) of high-grade adenomas), whereas the elevated type [3/5(60%)] predominated among cancerous lesions (Table 2).

**Table 2:** Macroscopic appearance of the duodenal neoplasms observed using the endocytoscopy system.

	Low-grade adenoma	High-grade adenoma	Cancer
Diameter (mm)	5 (2–12)	7 (3–40)	20 (7–50)
**Macroscopic appearance**			
Elevated	6	2	3
Flat	23	5	1
Depressed	3	2	1
Total	32	9	5

### ECS findings

**Figure 3:**
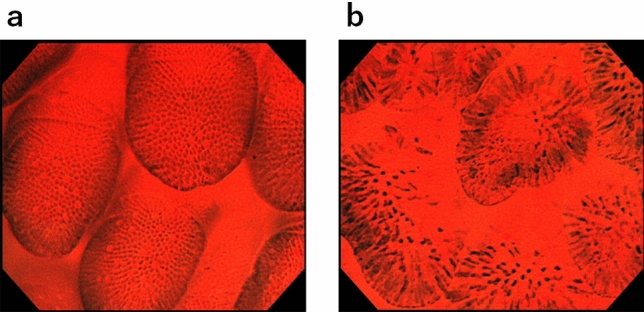
Endocytoscopic appearance of the normal duodenal mucosa. Pictures obtained using the maximum magnification power of the ECS (×500). (**a**) Normal duodenal villi 1 min after staining with methylene blue. Cells show a regular arrangement in each villus with sparse goblet cells. (**b**) Approximately 10 min later, goblet cells have become inconspicuous, and spindle-shaped nuclei have become evident.

In normal duodenal mucosa, cells were observed to have a regular arrangement in each villus using the maximum magnification of the GIF-H290EC at about one minute after the dye had been sprayed. Sparse goblet cells were evident (Fig. 3a) at the early phase of staining. With increased time, goblet cells became inconspicuous, and spindle-shaped nuclei became evident (Fig. 3b).

**Figure 4:**
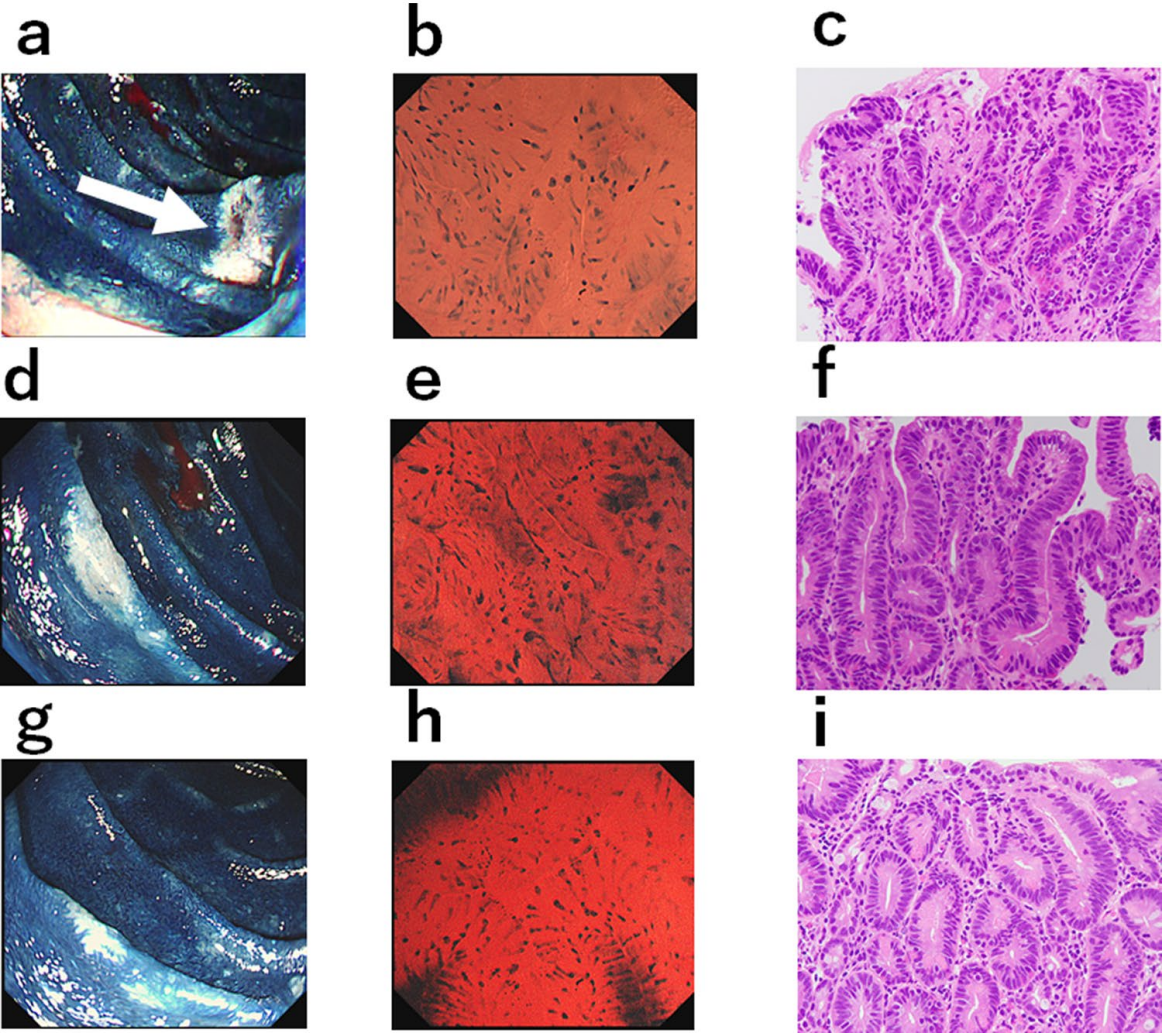
Multiple duodenal neoplasms in a familial adenomatous polyposis patient. All lesions were observed in the same patient. (**a**) A 7-mm flat-type unstained area (arrow). (**b**) Maximum magnification of the ECS (× 500) reveals some enlarged oval-shaped nuclei admixed among spindle-shaped nuclei. (**c**) Histologically, this lesion was diagnosed as duodenal cancer (×400). (**d**) A 7-mm flat-type unstained area. (**e**) ECS at maximum magnification (×500) reveals some enlarged oval-shaped nuclei admixed among spindle-shaped nuclei. (**f**) Histologically, this lesion was diagnosed as high-grade tubulovillous adenoma (×400). (**g**) A 5-mm flat-type unstained area. (**h**) ECS at maximum magnification (×500) reveals only spindle-shaped nuclei with duct openings. (**i**) Histologically, this lesion was diagnosed as low-grade tubular adenoma (×200).

In all five of the duodenal cancer lesions, enlarged oval nuclei were confirmed to be scattered among the spindle-shaped nuclei (Fig. 4a–c). Three of 9 high-grade adenomas (33.3%) and 3 of 31 (9.4%) low-grade adenomas were confirmed to have enlarged oval nuclei (Fig. 4d–f). The remaining 35 duodenal adenomas showed only spindle-shaped or non-enlarged oval nuclei (Fig. 4g–i, Table 3). Considering that enlarged oval nuclei are a feature of duodenal cancer, the sensitivity, specificity, positive predictive value, negative predictive value, and overall accuracy were 100% (5/5), 85.4% (35/41), 45.5% (5/11), 100% (35/35), and 87.0% (40/41), respectively.

**Table 3:** Shape and size of the nucleus observed using the endocytoscopy system.

Shape of the nucleus	Nuclear enlargement	Low-grade adenoma	High-grade adenoma	Cancer
Admix of oval nuclei	+	3	3	5
	−	2	1	0
Spindle nuclei only	+	5	2	0
	−	22	3	0
Total		32	9	5

Among the 41 histologically proven tubular or tubulo-villous adenomas, duct openings were observed in 37 (90.2%). In addition, finger-like projections, i.e. pointed villous structures, were observed in 15 of the 17 tubulovillous adenomas (88.2%), but in only 8 of the 24 tubular adenomas (33.3%) (Table 4).

**Table 4:** Microstructure of duodenal adenoma observed using the endocytoscopy system.

	Tubular adenoma (n = 24)	Tubulovillous adenoma (n = 17)
Duct opening	21 (87.5%)	16 (94.1%)
Finger-like projection	8 (33.3%)	15 (88.2%)

## Discussion

It has been reported previously that NBI endoscopy with or without magnification can detect more adenomatous lesions than WL endoscopy^8^. In the present study, we found that MB staining was able to detect more duodenal neoplasms than conventional WL observation and image-enhanced endoscopy using NBI. The score for the number of adenomatous lesions based on the Spigelman classification increased significantly in the order WL < NBI < MB staining. It is unclear why adenomas are observed as unstained lesions after MB staining. One possible explanation may be a difference in the degree of MB absorption between the normal duodenal mucosa and duodenal neoplasms. In the normal duodenal mucosa, all normal cells were stained by MB, whereas only sparse neoplastic cells were stained. This difference in staining may cause a visually evident difference in color. Furthermore, duodenal neoplasms are often observed as milky whitish because the duodenal adenoma and cancer cells retain lipid droplets^13^. For these reasons, milky whitish duodenal neoplasms may be clearly enhanced after MB staining, relative to the blue-stained normal duodenal mucosa. In the present study, more than 90% of the histologically examined lesions among areas unstained by MB were proven to be duodenal neoplasia histologically. This suggests that MB can be used for early detection of duodenal neoplasms, similar to iodine staining for detection of esophageal cancer.

According to the original Spigelman classification, FAP patients at stage V or IV are recommended to undergo surgical resection of the duodenum. Recently, the use of pancreas-preserving total duodenectomy, which is considered less invasive than conventional pancreato-duodenectomy, has been reported from some limited institutions^14^. Although the most preferable approach for duodenal neoplasms in FAP patients would be endoscopic treatment and preservation of the duodenum^15,16^, the incidence of duodenal cancer is considerably low, and over-treatment of duodenal adenomas with low malignant potential should be avoided. In this situation, efficient selection of adenomas with high malignant potential requiring endoscopic treatment is mandatory for patients with duodenal polyposis. A reddish color and a maximum diameter of 5 mm or 10 mm are reported to be predictive indicators of duodenal cancer^17,18^. In the present study, however, we were unable to distinguish between low-grade and high-grade adenoma on the basis of maximal diameter because the smallest high-grade adenoma was 3 mm and the largest low-grade adenoma was 12 mm. Our study results indicated that elevated-type (27.3%) and depressed-type (16.7%) lesions were more likely to be cancerous than flat-type lesions (3.4%).

The surface mucosal pattern and microvascular pattern revealed by NBI magnification endoscopy are reported to be useful for distinguishing duodenal adenoma from duodenal cancer^13^. The use of ECS adds additional cellular information in real time to the findings of NBI magnification endoscopy in vivo. We previously reported that “enlarged oval-shaped nuclei” are a characteristic feature of duodenal cancer when using the maximum magnification power of the ECS^12^. In the present study, enlarged oval nuclei were confirmed in all of the duodenal cancer lesions. In addition, 33.3% of high-grade adenomas also contained enlarged oval nuclei, compared with 9.4% of low-grade adenomas. Thus, the presence of “enlarged oval nuclei” may be a good indicator for endoscopic treatment. In addition, we found that ECS was able to predict structural atypia. Duct openings were evident in more than 90% of tubular or tubulovillous adenomas. Finger-like projections were observed in 88.2% of tubulovillous adenomas, but in only 33.3% of tubular adenomas. Intermixing of villous structures is considered to be a sign of high malignant potential in the Spigelman classification. Thus, finger-like projections observed by ECS may also be an indicator of the need for endoscopic treatment. Considering these findings as a whole, for surveillance examination of the duodenum in FAP patients, ECS observation using methylene blue staining provides more detailed information. Macroscopically evident elevated and depressed lesions should be the focus of attention. In addition, endoscopic resection should be considered for any lesions with enlarged oval-shaped nuclei or with villous structures evident by ultra-high magnifying observation using ECS. Adequate selection and endoscopic resection for lesions with high malignant potential in FAP patients will help achieve preservation of the duodenum.

Other than those mentioned above, ECS has two additional diagnostic advantages for observation of duodenal lesions in FAP patients. First, histological diagnosis using ECS in real time may make it possible to omit biopsy histology. The use of endoscopic forceps for biopsy sampling of tissue from duodenal lesions sometimes induces severe fibrosis, making subsequent endoscopic treatment difficult^19^. For this reason, it would be of help to endoscopists if duodenal adenomas with a high possibility of malignancy could be selected using ECS. Second, it has been reported that duodenal biopsy sampling has a relatively low accuracy (71.6%)^20^. Because duodenal cancer arises from duodenal adenoma (via the adenoma-carcinoma sequence)^21^, any cancerous region within the tumor may be limited. This makes it difficult for endoscopists to obtain adequate biopsy samples, and in this context the use of ECS might increase the accuracy of endoscopic diagnosis.

However, ECS observation of the duodenum in FAP patients has some limitations. First, a median examination time of 24 min is required. For this reason, we used conscious sedation for all patients. If ECS observation is attempted simultaneously with endoscopic resection, general anesthesia should be applied to minimize patient discomfort. Second, all examinations were performed by an experienced endoscopist. Some technical difficulties caused by peristalsis were experienced, making it essential to use intravenously injected butyl scopolamine to arrest mucosal movement. Third, the present study was retrospective, conducted at a single center, and included only a limited number of cases. Furthermore, the final histological diagnosis for the majority of the polyps included in this study was made using biopsy samples. Histological diagnosis using biopsy samples cannot be as informative as that using resected specimens. Therefore, accumulation of further FAP cases and more detailed histological evaluation in a multi-center setting will be necessary. Fourth, MB can cause oxidative damage to DNA when photosensitized by white light^22^. The accompanying risk needs to be carefully balanced against the possible benefit of improved early detection of preneoplastic lesions with MB chromoendoscopy.

In conclusion, MB staining allows more accurate detection of duodenal neoplasms in comparison with conventional WL and NBI observation. Use of the maximum magnification power of the ECS may facilitate selection of lesions with high malignant potential in patients with FAP.
